# Rural transport and climate change in South Africa: Converting constraints into rural transport adaptation opportunities

**DOI:** 10.4102/jamba.v11i3.718

**Published:** 2019-07-03

**Authors:** James Chakwizira

**Affiliations:** 1 Department of Urban and Regional Planning, University of Venda, Thohoyandou, South Africa

**Keywords:** Rural Transport, Climate Change, Impacts, Interventions, Framework, South Africa.

## Abstract

This study explored the implications of climate change for rural transport in South Africa. The article was seeking to convert existing rural transport adaptation constraints into rural transport adaptation opportunities. Challenges and constraints to rural transport adaptation transitions were also explored. The research methodology adopted was a review of the literature and references to case study examples. Then a four-stage multi-analytical approach was used to unravel and decode the major rural transport and climate change issues in South Africa. Consequent to the analysis, a framework of analysis for strongly integrating climate change to rural transport interventions was advanced. The findings indicated the existing rural transport adaptation measures and options in South Africa. The article concludes by highlighting the complexity and intricate dynamic nature of interactions, networks and systems that impact rural South Africa. Recommendations revolve around properly situating rural transport and climate change within the wider rural development challenges and matters facing contemporary South Africa.

## Introduction

Climate change adaptation in South Africa is one of the critical ways in which South Africa seeks to transition to a low-carbon economy (SAEON 2011). It is acknowledged that climate change in South Africa presents projected significant potential impacts on the ‘circular economy’ with significant implications for the growth and development trajectory of the country (Department of Environmental Affairs and Planning Western Cape 2008). Climate change foresight studies highlight the cross-cutting impact of the phenomenon on sectors of the economy (AfDB 2012a; IPCC 2001, 2007a). The rural transport sector will need to adapt to climate change agenda. The generic climate change impacts (which will require both generic transport responses complemented by specific actions) include the following:

- an increase in air temperature of 1°C–3°C over the next three to five decades
- an expected increase in evapotranspiration by 5% – 15% by 2050 in Southern Africa, with concerns for available water resources in the country
- challenges for agricultural production in both the summer and winter rainfall areas by mid-century, with particular concern for the maintenance of rural livelihoods
- changes in the territorial range and prevalence of a variety of vector-borne diseases such as malaria and schistosomiasis (bilharzia), as well as a potential increase in the spread of food- and water-borne diseases
- an increase in the frequency and intensity of extreme weather events such as flooding and wild fire, with heightened requirements for effective disaster management. (IPCC 2007b; Midgley et al. 2007)

Indeed, South Africa is rapidly urbanising with an urban population of 62% and a rural population of 38% (Stats SA, Census – 2011). The urbanisation (i.e. 2.85% average urban population increase in South Africa for the period 1996–2011) and migration trends (i.e. average of 22% for the period 1996–2011) have spatial implications for the provision of rural and urban transport (StatsSA 2011). Understanding the urbanisation dividend in the context in which rural transport climate change impacts and transitions are presenting new constraints and opportunities for the sector is important. Exploring how rural transport and climate change impacts play out in reality is important in creating action space for interventions. The rural transport and climate change implications discussion is conducted in a context in which the interdependencies between rural and urban areas are acknowledged. The built and non-built environment (in both urban and rural areas) can be viewed as ‘machines’ and ‘tools’ in managing and controlling climate change impacts. Although a green transport strategy (2016–2021) has been developed by the Department of Transport, the downside of the green transport policy is that it inadequately tackles the rural transport and climate change adaptation dimension in South Africa (Department of Transport 2016). Climate change adaptation with respect to transportation is an urgent matter in South Africa, given that we have rural infrastructure and communities that are already threatened by their situation on unsuitable sites, for example, housing developments on sites vulnerable to flooding or coastal infrastructure below the high water mark (Pinstrup-Andersen & Shimokawa 2010; Schweikert et al. 2014).

### Aim and objectives

The main aim of this article was to investigate the implications of climate change on rural transport in South Africa. The main aim is achieved through answering the following objectives:

1. to explain the implications of climate change on rural transport systems, operations and services in South Africa
2. to assess how the Sustainable Development Goals (SDGs) can be domesticated for enhanced rural transport performance in South Africa
3. to examine the constraints that impede the realisation of a ‘seamless’ rural transport transition to a low-carbon economy in South Africa
4. to recommend rural transport and climate change adaptation actions and measures aimed at (re)solving identified issues.

The following questions were generated to ensure that the article’s aim and objectives could be achieved:

1. How does climate change affect rural transport systems, operations and services in South Africa?
2. How can the SDGs agenda be domesticated for enhanced rural transport performance in South Africa?
3. Which constraints, if any, exist regarding rural transport transition to a low-carbon economy in South Africa?
4. Which solutions can be proffered in seeking to mainstream and strongly foster a sustainable rural transport and climate change agenda in South Africa?

## Research methodology

This study presents the results of a predominantly desktop-based research inquiry into rural transport and climate change in South Africa. Both published and grey literature were analysed. References to case studies were used in deepening the analysis. The four-stage multi-analytical approach used in the study was developed from the framework method (Ritchie & Lewis 2003). The stages involved were:

1. Critical review of transport and climate change documents and literature in the public domain: the intention of this review was to analyse the literature in search of common themes and categorise them by sub-themes such as transport strategies, policies and standards, accessibility and mobility, climate change and adaptation (Pope & Mays 2009).
2. Carrying out a systematic search for patterns to generate full descriptions of the rural transport and climate change matters that were capable of shedding light on the phenomena under investigation (Crotty 1988).
3. Application of inductive and deductive thematic analysis of rural transport and climate change issues identified (Hsieh & Shannon 2005; Polit & Beck 2010:1453).
4. Engaging in critical reflection on rural transport and climate change in order to draw conclusions and advance recommendations for overcoming identified obstacles and constraints.

A few selected transport and climate change experts were consulted as a way of supplementing the literature and providing a contextual interpretation of findings and implications to South Africa.

### Research limitations

Although the study explored the rural transport and climate change implications in South Africa, it did not look at the detailed manifestations of rural transport and climate change in South Africa. The article is therefore essentially a succinct resource on rural transport and climate change. However, the following matters are identified as areas for future research.

Exploring rural transport and climate change priorities is an area requiring further investigation. This study would be aimed at improving the understanding of climate change impacts and how they impact livelihoods (Nhemachena 2009; Nhemachena & Hassan 2007). At the same time, there will be a need to validate climate change models and response strategies through long-term environmental observation and research systems. There is also a need for in-depth studies that quantify the costs of climate-related impacts on transportation for planning and adaptation measures (DEA 2015; Madzwamuse 2010). There is also scope for joint publications through fostering collaboration among social, biological and physical sciences and recognising the role and opportunities for all sectors of society in contributing towards enhanced mitigation and adaptation of climate-related impacts on transportation. Although the study touched on aspects related to the need to improve transportation, climate change communication did not do this exhaustively.

There is also a need to conduct parallel studies examining the impact of the shift to active transport (walking and cycling), and rapid transit and public transport combined with improved land use can yield much greater immediate health ‘co-benefits’ than improving fuel and vehicle efficiencies especially in rural areas (Stats SA 2014; Von der Heyden, Hastings & Leitner 2014; World Bank 2016:1). Such rural transportation strategies need more systematic analysis in the assessment of transport mitigation measures in South Africa.

Transportation experts in South Africa should also consider health co-benefits more systematically (and potential risks) of transport mitigation strategies to highlight policies with the greatest overall gains for society, which was not implicitly focused by this article.

## Literature review

### Climate change implications on rural transport in South Africa

This section, making use of the literature review and the gap technique, discusses the implications of climate change on rural transport in South Africa.

### Rural transport and climate change

Climate change is a major threat to sustainable development in South Africa (SAEON 2011). Inherently, climate change poses one of the greatest threats towards the achievement of the SDGs. This is because South Africa is one of the highest Greenhouse Gas (GHG) emitting nations in the African continent (i.e. an average of 8.9 tonnes per capita) (Olivier et al. 2016). Greenhouse gas emissions in South Africa mainly emanate from the industrial and mining hubs. These emissions are driven by a sharp increase in fossil fuel use. At the same time, South Africa is highly vulnerable to climate change impacts, including more frequent droughts, floods and other extreme events and related reductions in agricultural productivity, accessibility and mobility constraints, threats to food security and risks of conflict over scarce land and water resources (refer to works by Nhemachena 2009; Nhemachena & Hassan 2007). This context highlights the compelling need to explore the climate change narrative meaning for rural transport in South Africa.

### Rural transport infrastructure vulnerability in South Africa

The optimum development, improvement and sustainability of rural infrastructure are vital for rural economic transformation, growth and development (Pinstrup-Andersen & Shimokawa 2010). This is in terms of the following:

- reducing the input costs and transport costs to the markets
- increasing and expanding the market size for farmers
- facilitating increased trade flows between areas, regions and countries
- incentivising value addition, encouraging innovation and spurring competitiveness
- promoting the crowding in of investments that is important in changing the spatial economic structure of an area, region and country.

Inherently, an inefficient transport system presents multiple constraints for the rural agriculture sector. This is in terms of raising the cost inputs into the production process as well as through delaying the sale of harvested crops (Fan 2004). Furthermore, investment in infrastructure is essential to stimulate the rural non-farm economy and (re)vitalise rural towns, as well as to facilitate the integration of marginal, peripheral and disadvantaged rural areas into national and international economies (Pinstrup-Anderson & Shimokawa 2010). Table 1 shows sub-Saharan Africa’s infrastructure needs by sector for 2006–2015.

**TABLE 1:** Sub-Saharan Africa’s infrastructure needs by sector (2006–2015).

Region	US$ Billion a year	GDP share (%)	Water supply and sanitation	Energy	ICT	Transport

	Year shares					
Middle income	17.92	6.62	4.89	80.93	0.95	13.23
Oil exporting	18.73	8.97	16.84	41.97	3.14	38.05
Low-income countries (non-fragile)	24.15	21.4	16.87	48.42	3.54	31.17
Low-income countries (Fragile)	16.38	42.92	10.96	56.99	2.34	29.71
Africa	74.9	11.69	13.39	56.9	2.57	27.14

*Source*: African Development Monitor (2012)

GDP, gross domestic product; ICT, information and communication technology.

From Table 1, we can deduce that there is a huge rural infrastructure backlog resulting from decades of underinvestment, lack of maintenance and destruction and dilapidation because of conflict (African Development Monitor 2012). The need to improve the quality of infrastructure, which is generally poor and which is part and parcel of climate change adaptation, is therefore important.

### Overview of Rural accessibility Index in South Africa

According to the Rural Accessibility Index (RAI), only 34% of the African rural population lives within 2 km of rural roads, compared with 90% in East Asia and the Pacific countries, as well as 59% in Latin America (African Development Monitor 2012). Table 2 shows a summary of the RAI for a select few countries in the world.

**TABLE 2:** Access to rural transport measured by total population and Rural Access Index.

Country	People without access to rural transport (in millions)	Rural Access Index
India	301	61
China	23.5	97
South Africa	14.8	21
Brazil	14.2	53
Mexico	9.7	61
USA	8.2	86
Russia	7.4	81
Germany	2.3	89
Saudi Arabia	1.1	75
Japan	0.5	99
France	0.2	99
United Kingdom	0.2	99

*Source*: World Bank (2009)

From Table 2, we can deduce that the rural transport index in South Africa is poor, at approximately 21%. The need to improve the RAI of South Africa as a climate change adaptation measure is critical. This is because improved RAI infrastructure and services supplement climate change-resilient infrastructure and measures in any area (African Development Monitor 2012). Furthermore, in South Africa, during the poverty hearings held in 2008, the lack of roads, telecommunication and access to health facilities were also mentioned as major stumbling blocks in rural areas in Limpopo and the Northern and Eastern Cape provinces. Communities in these regions reflected that they often had to travel long distances to access basic facilities (EPWP 2009; ILO/EPWP 2007).

### The food miles debate

Climate change within the transport domain has ushered in the emergence of the ‘food miles debate’ (McKinnon et al. 2015). This debate revolves around questioning the ‘transport distance that food products travel from production site to consumption market or food outlet’ (Weber & Matthews 2008). This is much acute in a global village in which ‘the food plate debate’ is strongly linked to the GHGs emitted as well as the need to raise new social consciousness. The use of air and road transport has added to the complexity in exploring the carbon dioxide emissions, which contribute to climate change. Understanding the ‘food miles’ debate is important for South Africa’s rural areas, as climate change transportation implications have a direct bearing on the evolving rural agricultural systems and economies.

### Department of Transport’s Rural Transport Strategy (2007)

The Rural Transport Strategy (RTS) was crafted with the intention of developing balanced and sustainable rural transport systems through supporting local infrastructure and services. The RTS clearly articulates the need to improve access roads, developing passable roads and addressing neglected infrastructure and corridors, which are linked to markets and other social services. However, its downside was that it did not consider climate change.

### Linking Rural Access Index and the Sustainable Development Goals

The SDGs have gone a step further than the Millennium Development Goals in making an explicit provision and recognition of the role that transport plays in development – be it in urban or rural spaces but most importantly in facilitating the realisation of all other SDGs (SLOCAT 2015). Transport is instrumental in advancing goals on food security, health, energy, infrastructure development, urban development, sustainable consumption and production as well as climate change (World Bank 2009). The fact that transport-related targets are included explicitly and implicitly in at least a minimum of 7 out of the 17 SDGs illustrates the cross-cutting role that transport has in sustainable development. Sustainable transport is indeed a cross-cutting thematic area and cross-sectional issue that shapes and is shaped by the developmental trajectory of past, present and future generations alike.

Although the transport acknowledgement in the SDGs is much clear in the context of urban infrastructure and resilience in cities (which is however not enough), this is however viewed as a good starting point. Similar inroads regarding the acknowledgement and strategic positioning and leadership role being granted for rural transport will lead to a complete inclusion of transport in seeking to promote and support the realisation of the SDGs. As an example, Target 9.1 of the SDGs seeks to develop quality, reliable, sustainable and resilient infrastructure, including regional and trans-border infrastructure, to support economic development and human well-being, with a focus on affordable and equitable access for all. In the short term, enhancing one aspect of decent infrastructure (i.e. rural road connectivity) reduces transport costs and improves access to markets and social facilities such as schools and hospitals. In the longer term, it elevates agricultural productivity, business profitability and employment, which reflects the aims of SDG Target 2.3 – doubling the agricultural productivity and incomes of small-scale food producers. Better connectivity also strengthens the resilience of rural populations to natural and human-made shocks and disasters by facilitating the movement of people and supplies for faster recovery (IPCC 2001).

### Towards a conceptual framework in understanding rural transport and climate change in South Africa

**FIGURE 1:**
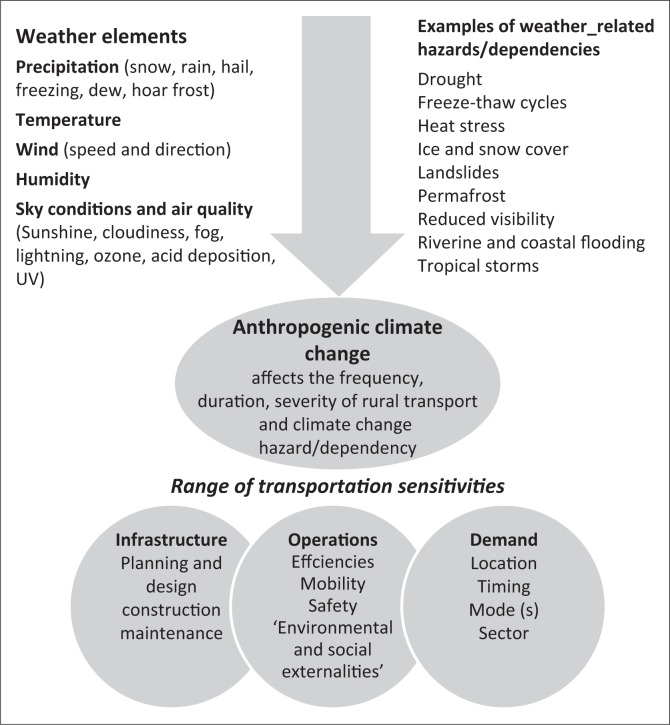
A conceptual framework for studying climate change and rural transport in South Africa.

Figure 1 shows a conceptual framework towards understanding rural transport and climate change in South Africa.

As shown in Figure 1, we can deduce that rural transport and climate change dynamics are a complex phenomenon. Generating appropriate interventions and policy actions requires co-ordinated and collaborative efforts from multi-disciplinary teams comprising professionals from engineering, community development, economics, accounting, town planning, geography, political administration and the environmental fields.

## Research analysis and findings

### Constraints that impede the realisation of seamless rural transport transition to a low-carbon economy in South Africa

#### Climate change impacts in rural areas

The main challenges likely to face African populations will emanate from the effects of extreme events such as tropical storms, floods, landslides, wind, cold waves, droughts and abnormal sea-level rises that are expected as a result of climate change (AfDB 2012b). These events are likely to exacerbate management problems relating to pollution, sanitation, waste disposal, water supply, public health, infrastructure and technologies of production (IPCC 1996). In this regard, the following interventions are essential:

- Effective connections are essential for enabling local transport networks.
- Roads, paths, bridges and waterways need to link people with markets and services, such as education and health clinics.
- Fields and plantations must be accessible for farmers and producers and agricultural produce must be able to be transported from the field to the market.

#### Climate change and rural transport

The effect of climate change will lead to roads and paths being flooded more frequently. This will impact mobility and accessibility to goods and services. Roads in flat areas are at great risk from simple flooding, and roads in mountainous areas are more at risk of damage from the actions of rapidly flowing water in the drains and in cross streams. Both see communities becoming cut off and journeys taking much longer. At the very least, this leads to problems in transporting crops to markets and delivering agricultural inputs to villages. It will also lead to fewer visits being made by clinic staff to all sections of the community and to increased absenteeism from school. Even the collection of safe drinking water and fuel wood can become more difficult.

### Rural transport and climate change in South Africa (the road dimension)

In rural areas, roads represent a lifeline for economic and agricultural livelihood, as well as a number of indirect benefits including access to healthcare, education, credit, political participation and more (Sayeg, Starkey & Huizenga 2014). Roads may be sparse through geographic locations, making each road critical (every road matters and counts). Extreme events pose a costly hazard to roads in terms of degradation, necessary maintenance and potential decrease in lifespan because of climatic impacts. Climate change poses costly impacts in terms of maintenance, repairs and lost connectivity (Schweikert et al. 2014:306).

### Adaptation through improving rural transport

The raising of the levels of roads and paths is a straightforward solution to the problem, provided that thought is given to sufficient culverts, channels and other drains that permit the water to drain away (Gordon & Sherar 2003). The planning and design of new rural roads and paths must also take into account future rainfall and flood levels. Where possible, consideration should be given to combining transport and flood control benefits from a raised road embankment. Where a new raised path is being built, it may be sensible to pave it to protect it from heavy rain so that bicycles and motorcycles can use it in all weathers. The building of new footbridges along such raised paths will be important and is within the capacity of communities provided they receive technical support.

### Local resource-based approaches

The local resource-based method emphasises the use of local capacities throughout the whole process of the transport infrastructure development. The approach applies not only to the use of local labour and materials, but also to local-level planning, the use of local contractors and community groups for implementation of infrastructure works. Moreover, local communities can be encouraged to make use of simple means of transport based on bicycles, motor cycles and single-axle tractors. The local resource-based approach has been applied, with support from the International Labour Organisation (ILO), in several countries in the region. Experience shows that these approaches provide social and environmental benefits, at the same time reducing the cost of road rehabilitation and maintenance (Gordon & Sherar 2003).

### Climate change impacts in Western Cape: A case study

The rural transport sector in the Western Cape province is already under stress, and development planning has negatively impacted the sector in that communities are being established further and further away from economic nodes (Department of Environmental Affairs and Planning, Western Cape 2008). The sector is a significant contributor to provincial GHG emissions, and growth in demand will exacerbate this problem. In addition, local air quality is impacted by transport. Options for mitigation in the sector include introducing cleaner fuel programmes in the provincial fuel mix and commercialising innovation in the province such as the development of South Africa’s first ‘home-grown’ electric car, being developed in Cape Town (www.2greenenergy.com). A bundle of measures is required to address the challenges. Options to adapt include integrating climate risks into development planning and approval processes; enhancing the emergency services and integrating climate risk into disaster management processes and systems; and maintaining livelihoods (such as rural livelihoods) as far as possible to minimise population stress in urban centres (World Bank 2009).

### Research contribution

**FIGURE 2:**
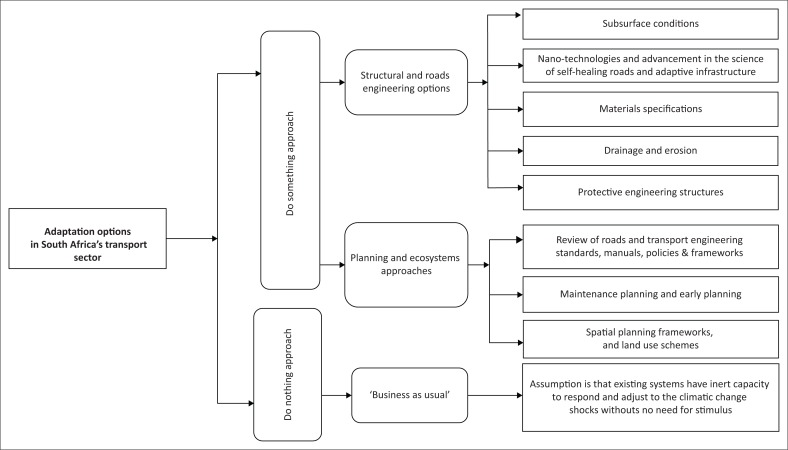
Schematic illustration of the rural transport adaptation options in South Africa.

Figure 2 shows a schematic illustration of the rural transport adaptation options in South Africa. It should be stressed that transport adaptation is an inter-related and dependent activity. For the solutions to stand the test of time, these initiatives must be complemented by similar adaptation responses from all sectors of the economy.

From Figure 2, it can be deduced that with regard to climate change impacts, some methods are proposed to adapt to climate change events, especially flooding, which always causes damage to rural roads.

### Ethical considerations

This article followed all ethical standards for a research without direct contact with human or animal subjects.

### Discussion and concluding remarks

A number of rural transport adaptation options and recommendations exist. These are briefly discussed in the section that follows.

### Protecting the rural road assets from climate change: Recommendations

- *Protecting the road infrastructure from the impacts of climate changes:* this can be achieved through ensuring that the road infrastructure does not increase the vulnerability of the surrounding area to climate change (Gordon & Sherar 2003). At the same time, providing early warnings and emergency management measures is also critical.
- *Improved planning for rural roads to accommodate climate changes:* this can be tackled through preparing vulnerability maps for rural roads as a result of climate change (World Bank 2009). At the same time, planning systems and institutions have to undertake improved planning for climate changes.
- *Identifying potential adaptation options:* once these have been identified, these should immediately be prioritised. It is also beneficial to conduct an economic analysis of climate-proofing measures to support the decision-making process (DEA 2015).
- *Reviewing the sustainability and capacity of existing rural transportation engineering designs, standards and guidelines:* there is an urgent need to review the current engineering designs, standards and guidelines to withstand climate changes, especially in rural areas, given the sharp twists in climatic regimes (Department of Transport 2016). The review should be followed by the development and implementation of training and updating of curriculums so that climate and transport adaptation-ready graduates are produced. In terms of the implementation and sustainability of rural transport surfacing options, the need to explore low-cost road surfacing guidelines is important. At the same time, new low-cost road surfacing technologies as indicated by non-technology, for example, should also be explored. Surface options for low-cost roads completion require the following issues to be addressed:
  - Low-cost structures: small structures for rural roads guideline
  - Low-cost slope stabilisation, eco-roads and surfacing, and rural footbridges
  - Road maintenance: general road maintenance retrospective – low-volume rural road maintenance and community maintenance.
- *Promoting green planning and infrastructure design, implementation and management:* there is a need to design and implement ecosystem-based adaptation strategies that focus on environmental or green planning for rural project roads (McKinnon et al. 2015). In addition, the planting of climate change resilient trees along project road embankments and select grass and biomaterials to improve flood and drought management (i.e. increasing ground cover and infiltration of floods and water retention during droughts) is essential. This should include the implementation of sustainable engineering infrastructure and services solutions. Such a solution will seek to promote and advance the deployment of environmentally optimised rural transport design standards and specifications.

## Conclusion

Climate change will impact rural transport, but a set of low-level transport solutions exist that can be implemented. Climate change impacts and implications on transport can be solved not only through transport solutions but also through non-transport solutions, for example, appropriate siting and location of schools, clinics, buildings, etc. Partnership and collaboration in addressing the complex and complicated challenges are very important. Reliable knowledge is necessary for effective policymaking; however, both knowledge generation and policymaking are continuous processes and neither can ever be complete (SAEON 2011:5). Sustainable rural development is contingent on responding to the risks posed by climate change. Rural transport plays a catalytic role in this equation and should be strategically improved and promoted for enhanced growth and development of the South African economy. To conclude, it is apt to quote DEA (2015):

In building climate resilience, it is important to select strategies that are either low cost and easy to implement, or strategies that are either win-win or no-/low-regret investment options (i.e. where the investment in the project will result in benefits regardless of the extent of climate change, and would not cause detriment).
